# *catena*-Poly[[(acetato-κ*O*)[4-(1*H*-pyrazol-3-yl)pyridine-κ*N* ^1^]zinc]-μ-acetato-κ^2^ *O*:*O*′]

**DOI:** 10.1107/S1600536811040190

**Published:** 2011-10-12

**Authors:** Zheng-De Tan, Feng-Jiao Tan, Bo Tan, Zhi-Wen Yi

**Affiliations:** a College of Chemistry and Chemical Engineering, Hunan Institute of Engineering, Xiangtan 411104, People’s Republic of China; b The People’s Hospital of Xiangtan County, Xiangtan 411104, People’s Republic of China

## Abstract

In the title compound, [Zn(CH_3_CO_2_)_2_(C_8_H_7_N_3_)]_*n*_, the Zn^II^ atom is coordinated by one N atom from a 4-(1*H*-pyrazol-3-yl)pyridine ligand and three O atoms from two bridging and one terminal acetate ligands, forming a distorted tetra­hedral geometry. The bridging acetate ligands link the Zn atoms into a chain along [001]. N—H⋯O hydrogen bonds and π–π inter­actions between the pyridine and pyrazole rings [centroid–centroid distance = 3.927 (3) Å] connect the chains into a layer parallel to (011).

## Related literature

For background to complexes of 4-(1*H*-pyrazol-3-yl)pyridine, see: Davies *et al.* (2005 ▶). For the synthesis of the ligand, see: Davies *et al.* (2003 ▶).
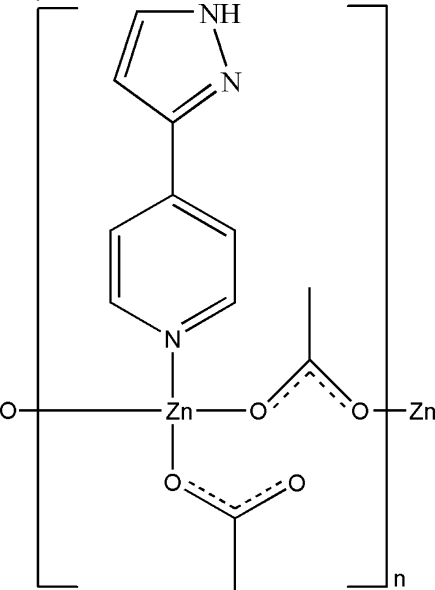


## Experimental

### 

#### Crystal data

[Zn(C_2_H_3_O_2_)_2_(C_8_H_7_N_3_)]
*M* *_r_* = 322.58
Monoclinic, 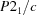

*a* = 16.371 (3) Å
*b* = 8.8526 (18) Å
*c* = 9.5041 (19) Å
β = 94.18 (3)°
*V* = 1373.7 (5) Å^3^
*Z* = 4
Mo *K*α radiation
μ = 1.80 mm^−1^
*T* = 293 K
0.28 × 0.23 × 0.19 mm

#### Data collection

Rigaku SCXmini CCD diffractometer
Absorption correction: multi-scan (*CrystalClear*; Rigaku, 2005 ▶) *T*_min_ = 0.632, *T*_max_ = 0.726
11822 measured reflections
2479 independent reflections
1957 reflections with *I* > 2σ(*I*)
*R*_int_ = 0.068

#### Refinement

*R*[*F* ^2^ > 2σ(*F* ^2^)] = 0.056
*wR*(*F* ^2^) = 0.131
*S* = 1.06
2479 reflections
181 parameters
H-atom parameters constrained
Δρ_max_ = 0.51 e Å^−3^
Δρ_min_ = −0.54 e Å^−3^

### 

Data collection: *CrystalClear* (Rigaku, 2005 ▶); cell refinement: *CrystalClear*; data reduction: *CrystalClear*; program(s) used to solve structure: *SHELXS97* (Sheldrick, 2008 ▶); program(s) used to refine structure: *SHELXL97* (Sheldrick, 2008 ▶); molecular graphics: *ORTEPII* (Johnson, 1976 ▶) and *DIAMOND* (Brandenburg, 1999 ▶); software used to prepare material for publication: *SHELXL97*.

## Supplementary Material

Crystal structure: contains datablock(s) I, global. DOI: 10.1107/S1600536811040190/hy2465sup1.cif

Structure factors: contains datablock(s) I. DOI: 10.1107/S1600536811040190/hy2465Isup2.hkl

Additional supplementary materials:  crystallographic information; 3D view; checkCIF report

## Figures and Tables

**Table 1:** Selected bond lengths (Å)

Zn1—N1	2.026 (4)
Zn1—O1	1.942 (4)
Zn1—O3	1.958 (3)
Zn1—O4^i^	1.984 (3)

Symmetry code: (i) 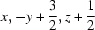.

**Table 2:** Hydrogen-bond geometry (Å, °)

*D*—H⋯*A*	*D*—H	H⋯*A*	*D*⋯*A*	*D*—H⋯*A*
N3—H3⋯O2^ii^	0.86	1.93	2.769 (6)	163

Symmetry code: (ii) 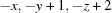.

**Fig. 1.**
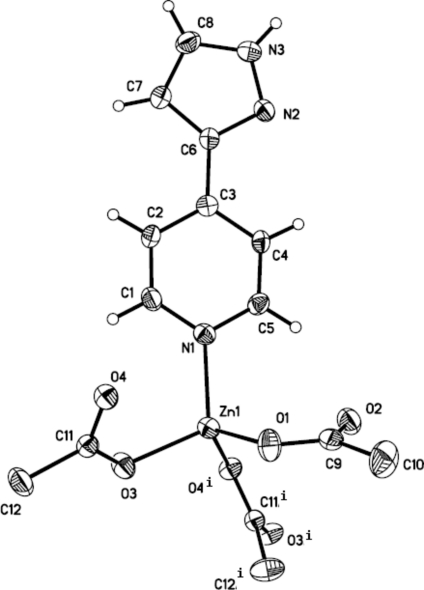
The asymmetric unit of the title compound. Displacement ellipsoids are drawn at the 30% probability level. [Symmetry code: (i) x, 3/2-y, 1/2+z.]

**Fig. 2.**
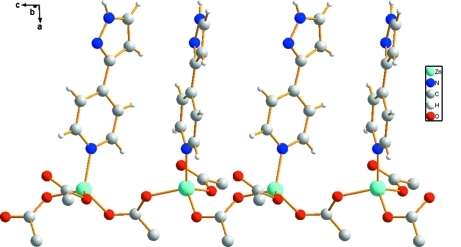
A view of the one-dimensional structure of the title compound along [0 0 1].

**Fig. 3.**
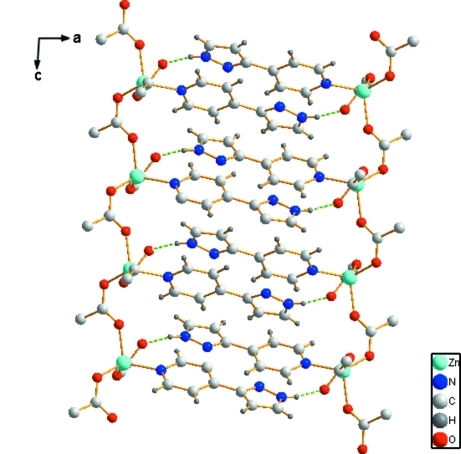
A view of the layer network. Hydrogen bonds are shown as dashed lines.

## supplementary crystallographic information

### Comment

Pyridine derivatives are an important class of ligands for constructing metal–organic frameworks. 4-(1*H*-Pyrazol-3-yl)pyridine can be used as pyridine ligand in building coordination compounds (Davies *et al.*, 2005). In the present paper, we report the synthesis and structure of the title compound.

As shown in Fig. 1, the Zn^II^ atom exhibits a distorted tetrahedral coordination geometry, defined by one N atom from a 4-(1*H*-pyrazol-3-yl)pyridine ligand and three O atoms from two types of acetate ligands (Table 1). One acetate anion coordinates the Zn atom as a monodentate terminal ligand. The other acetate anion links the Zn atoms *via* two O atoms, forming a one-dimensional chain along [0 0 1] (Fig. 2). N—H···O hydrogen bonds (Table 2) and π–π interactions between the pyridine and pyrazole rings [centroid–centroid distance = 3.927 (3) Å] connect the chains into a layer parallel to (0 1 1) (Fig. 3).

### Experimental

4-(1*H*-Pyrazol-3-yl)pyridine was prepared according to the published method of Davies *et al.* (2003). An aqueous solution (20 ml) containing zinc acetate (0.1 mmol, 22 mg) and 4-(1*H*-pyrazol-3-yl)pyridine (0.2 mmol, 29 mg) was stirred for a few minutes in air. Colorless crystals were obtained by allowing the solution to stand at room temperature for a few weeks.

### Refinement

H atoms were placed at calculated positions and refined as riding atoms, with C—H = 0.93 and N—H = 0.86 Å and with *U*_iso_(H) = 1.2*U*_eq_(C, N).

### Crystal data

**Table:**

[Zn(C_2_H_3_O_2_)_2_(C_8_H_7_N_3_)]	*F*(000) = 648
*M**_r_* = 322.58	*D*_x_ = 1.560 Mg m^−^^3^
Monoclinic, *P*2_1_/*c*	Mo *K*α radiation, λ = 0.71073 Å
Hall symbol: -P 2ybc	Cell parameters from 11614 reflections
*a* = 16.371 (3) Å	θ = 3.2–27.6°
*b* = 8.8526 (18) Å	µ = 1.80 mm^−^^1^
*c* = 9.5041 (19) Å	*T* = 293 K
β = 94.18 (3)°	Block, colourless
*V* = 1373.7 (5) Å^3^	0.28 × 0.23 × 0.19 mm
*Z* = 4	

### Data collection

**Table:**

Rigaku SCXmini CCD diffractometer	2479 independent reflections
Radiation source: fine-focus sealed tube	1957 reflections with *I* > 2σ(*I*)
graphite	*R*_int_ = 0.068
ω scans	θ_max_ = 25.2°, θ_min_ = 3.2°
Absorption correction: multi-scan (*CrystalClear*; Rigaku, 2005)	*h* = −19→19
*T*_min_ = 0.632, *T*_max_ = 0.726	*k* = −10→10
11822 measured reflections	*l* = −11→11

### Refinement

**Table:**

Refinement on *F*^2^	Primary atom site location: structure-invariant direct methods
Least-squares matrix: full	Secondary atom site location: difference Fourier map
*R*[*F*^2^ > 2σ(*F*^2^)] = 0.056	Hydrogen site location: inferred from neighbouring sites
*wR*(*F*^2^) = 0.131	H-atom parameters constrained
*S* = 1.06	*w* = 1/[σ^2^(*F*_o_^2^) + (0.063*P*)^2^ + 1.9274*P*] where *P* = (*F*_o_^2^ + 2*F*_c_^2^)/3
2479 reflections	(Δ/σ)_max_ < 0.001
181 parameters	Δρ_max_ = 0.51 e Å^−^^3^
0 restraints	Δρ_min_ = −0.54 e Å^−^^3^

### Fractional atomic coordinates and isotropic or equivalent isotropic displacement parameters (Å^2^)

**Table:**

	*x*	*y*	*z*	*U*_iso_*/*U*_eq_	
Zn1	0.34131 (3)	0.68665 (6)	1.02084 (6)	0.0343 (2)	
N1	0.2233 (2)	0.7526 (5)	0.9820 (4)	0.0348 (9)	
C3	0.0573 (3)	0.8142 (5)	0.9123 (5)	0.0325 (11)	
C4	0.0820 (3)	0.6924 (5)	0.9969 (5)	0.0362 (11)	
H4	0.0430	0.6294	1.0325	0.043*	
C5	0.1635 (3)	0.6647 (6)	1.0280 (5)	0.0372 (12)	
H5	0.1784	0.5813	1.0836	0.045*	
C1	0.1992 (3)	0.8734 (6)	0.9045 (5)	0.0403 (12)	
H1	0.2393	0.9371	0.8736	0.048*	
C2	0.1192 (3)	0.9082 (5)	0.8682 (5)	0.0365 (12)	
H2	0.1059	0.9938	0.8146	0.044*	
N3	−0.1561 (3)	0.7919 (5)	0.8372 (5)	0.0491 (12)	
H3	−0.2025	0.7466	0.8393	0.059*	
C6	−0.0297 (3)	0.8401 (5)	0.8676 (5)	0.0331 (11)	
N2	−0.0849 (3)	0.7342 (5)	0.8937 (5)	0.0454 (11)	
C7	−0.0660 (3)	0.9619 (6)	0.7937 (5)	0.0438 (13)	
H7	−0.0402	1.0480	0.7624	0.053*	
C8	−0.1469 (3)	0.9277 (7)	0.7774 (6)	0.0495 (14)	
H8	−0.1880	0.9870	0.7333	0.059*	
O3	0.4195 (2)	0.8246 (4)	0.9418 (3)	0.0453 (9)	
O1	0.3590 (2)	0.4837 (4)	0.9518 (4)	0.0500 (10)	
O2	0.2878 (2)	0.3956 (4)	1.1226 (4)	0.0501 (10)	
C9	0.3292 (3)	0.3761 (6)	1.0208 (6)	0.0405 (12)	
C10	0.3494 (5)	0.2163 (7)	0.9709 (8)	0.072 (2)	
C11	0.4183 (3)	0.8446 (5)	0.8100 (5)	0.0334 (11)	
C12	0.4840 (3)	0.9438 (7)	0.7551 (6)	0.0578 (17)	
O4	0.3643 (2)	0.7841 (4)	0.7269 (3)	0.0373 (8)	

### Atomic displacement parameters (Å^2^)

**Table:**

	*U*^11^	*U*^22^	*U*^33^	*U*^12^	*U*^13^	*U*^23^
Zn1	0.0330 (3)	0.0415 (4)	0.0283 (3)	−0.0030 (3)	0.0026 (2)	−0.0012 (3)
N1	0.035 (2)	0.039 (2)	0.030 (2)	−0.004 (2)	0.0011 (18)	−0.0030 (19)
C3	0.034 (3)	0.034 (3)	0.030 (3)	0.003 (2)	0.005 (2)	−0.005 (2)
C4	0.039 (3)	0.034 (3)	0.036 (3)	−0.004 (2)	0.007 (2)	0.008 (2)
C5	0.040 (3)	0.041 (3)	0.030 (3)	0.003 (2)	0.003 (2)	0.006 (2)
C1	0.040 (3)	0.041 (3)	0.040 (3)	−0.010 (2)	0.004 (2)	0.003 (2)
C2	0.042 (3)	0.032 (3)	0.036 (3)	0.001 (2)	0.005 (2)	0.007 (2)
N3	0.032 (2)	0.053 (3)	0.061 (3)	−0.001 (2)	−0.002 (2)	0.007 (2)
C6	0.037 (3)	0.031 (3)	0.031 (3)	0.000 (2)	0.004 (2)	0.000 (2)
N2	0.034 (3)	0.039 (3)	0.063 (3)	−0.004 (2)	0.003 (2)	0.008 (2)
C7	0.045 (3)	0.043 (3)	0.043 (3)	−0.002 (3)	0.000 (2)	0.010 (3)
C8	0.042 (3)	0.054 (4)	0.052 (3)	0.009 (3)	−0.001 (3)	0.010 (3)
O3	0.048 (2)	0.061 (2)	0.0267 (19)	−0.0171 (18)	0.0011 (15)	0.0038 (17)
O1	0.068 (3)	0.038 (2)	0.046 (2)	−0.0065 (19)	0.0162 (19)	−0.0039 (17)
O2	0.044 (2)	0.046 (2)	0.062 (3)	−0.0055 (18)	0.0122 (19)	0.0023 (19)
C9	0.036 (3)	0.042 (3)	0.042 (3)	−0.004 (2)	−0.008 (2)	−0.001 (3)
C10	0.094 (5)	0.037 (4)	0.086 (5)	0.005 (3)	0.006 (4)	−0.013 (3)
C11	0.036 (3)	0.035 (3)	0.030 (3)	0.002 (2)	0.004 (2)	0.001 (2)
C12	0.047 (3)	0.075 (4)	0.052 (4)	−0.023 (3)	0.005 (3)	0.015 (3)
O4	0.042 (2)	0.044 (2)	0.0258 (17)	−0.0044 (16)	0.0040 (15)	−0.0009 (15)

### Geometric parameters (Å, °)

**Table:**

Zn1—N1	2.026 (4)	N3—C8	1.344 (7)
Zn1—O1	1.942 (4)	N3—N2	1.348 (6)
Zn1—O3	1.958 (3)	N3—H3	0.8600
Zn1—O4^i^	1.984 (3)	C6—N2	1.338 (6)
N1—C1	1.341 (6)	C6—C7	1.395 (7)
N1—C5	1.348 (6)	C7—C8	1.356 (7)
C3—C4	1.387 (6)	C7—H7	0.9300
C3—C2	1.398 (7)	C8—H8	0.9300
C3—C6	1.475 (7)	O3—C11	1.264 (6)
C4—C5	1.368 (7)	O1—C9	1.274 (6)
C4—H4	0.9300	O2—C9	1.233 (6)
C5—H5	0.9300	C9—C10	1.536 (8)
C1—C2	1.365 (7)	C11—O4	1.261 (5)
C1—H1	0.9300	C11—C12	1.510 (7)
C2—H2	0.9300		
			
O1—Zn1—O3	109.25 (16)	C3—C2—H2	120.2
O1—Zn1—O4^i^	115.59 (15)	C8—N3—N2	112.7 (4)
O3—Zn1—O4^i^	102.40 (14)	C8—N3—H3	123.6
O1—Zn1—N1	111.65 (17)	N2—N3—H3	123.6
O3—Zn1—N1	113.05 (16)	N2—C6—C7	111.5 (4)
O4^i^—Zn1—N1	104.60 (15)	N2—C6—C3	119.3 (4)
C1—N1—C5	116.5 (4)	C7—C6—C3	129.2 (4)
C1—N1—Zn1	124.6 (3)	C6—N2—N3	103.7 (4)
C5—N1—Zn1	118.7 (3)	C8—C7—C6	105.1 (5)
C4—C3—C2	116.7 (4)	C8—C7—H7	127.4
C4—C3—C6	121.4 (4)	C6—C7—H7	127.4
C2—C3—C6	121.8 (4)	N3—C8—C7	106.9 (5)
C5—C4—C3	120.2 (4)	N3—C8—H8	126.5
C5—C4—H4	119.9	C7—C8—H8	126.5
C3—C4—H4	119.9	C11—O3—Zn1	120.3 (3)
N1—C5—C4	123.1 (5)	C9—O1—Zn1	116.5 (3)
N1—C5—H5	118.4	O2—C9—O1	123.5 (5)
C4—C5—H5	118.4	O2—C9—C10	121.0 (5)
N1—C1—C2	123.9 (5)	O1—C9—C10	115.5 (5)
N1—C1—H1	118.1	O4—C11—O3	121.4 (4)
C2—C1—H1	118.1	O4—C11—C12	121.0 (4)
C1—C2—C3	119.5 (5)	O3—C11—C12	117.6 (4)
C1—C2—H2	120.2	C11—O4—Zn1^ii^	129.6 (3)

Symmetry codes: (i) *x*, −*y*+3/2, *z*+1/2; (ii) *x*, −*y*+3/2, *z*−1/2.

### Hydrogen-bond geometry (Å, °)

**Table:**

*D*—H···*A*	*D*—H	H···*A*	*D*···*A*	*D*—H···*A*
N3—H3···O2^iii^	0.86	1.93	2.769 (6)	163

Symmetry codes: (iii) −*x*, −*y*+1, −*z*+2.
